# Lymphoepithelioma-Like Carcinoma of the Bladder: A Case Report of a Rare and Particular Variant of Urothelial Carcinoma 

**DOI:** 10.1155/2018/7975454

**Published:** 2018-07-08

**Authors:** Youness Jabbour, Youssef Jabri, Hamza Lamchahab, Mohammed Tbouda, Ahmed Jahid, Tarik Karmouni, Khalid El Khader, Abdellatif Koutani, Ahmed Iben Attya Andaloussi

**Affiliations:** ^1^Urology B Department, Ibn Sina Teaching Hospital, Rabat, Morocco; ^2^Faculty of Medicine and Pharmacy, Mohammed V University, Rabat, Morocco; ^3^Anatomyical Pathology Department, Ibn Sina Teaching Hospital, Rabat, Morocco

## Abstract

Lymphoepithelioma-like carcinoma of the bladder (LELCB) is a rare variant of urothelial carcinoma first described by Zukerberg in 1991 and confirmed as a variant of urothelial carcinoma by the WHO classification of tumors of the urinary system. LELCB is characterized by a marked infiltration of lymphocytes in the area involved by the tumor which may coexist with the conventional urothelial carcinoma. LELCB are classified according to the percentage of lymphoepithelioma component within the tumor with the prognosis depending on the percentage. We report a new case of pure LELCB occurring in 63-year-old woman presenting with hematuria. Ultrasonography and cystoscopy revealed a large tumor on the left lateral wall of the bladder. Transurethral resection of the bladder tumor (TURBT) was performed. Pathological and immunohistochemical analysis revealed a high-grade muscle-invasive LELCB (G3pT2). The patient underwent an adjuvant systemic chemotherapy with no recurrence after a ten-month follow-up. To our knowledge, this is the second Moroccan case of LELCB reported in the English literature. Although its rare occurrence prognosis and ideal therapeutic management of LELCB have not been clearly established yet, literature findings encourage the adoption of a conservative approach in treatment of LELCB.

## 1. Introduction

Bladder cancer ranks as the ninth most frequently diagnosed cancer worldwide in 2012 with a male predominance. Its incidence is stabilizing or declining in men while some increasing trends are seen for women [1].

This observed evolution of bladder cancer incidence appears to reflect the recently growing contact of women with the causes of bladder cancer such as tobacco and occupational exposures.

The most common histological type of bladder cancer is urothelial carcinoma which constitutes more than 90% of all bladder cancers.

Urothelial carcinoma has been known to have a remarkable propensity for divergent differentiation which is seen most commonly in association with high-grade and locally advanced disease and which histology is associated with features of biologically aggressive disease [2].

The fourth edition of the WHO classification of tumors of the urothelial tract listed ten variants of urothelial carcinoma differentiation [3].

Lymphoepithelioma-like carcinoma (LELC) is a rare variant of infiltrating urothelial carcinoma first described in the bladder by Zukerberg et al. in 1991 [4].

We herein report a new case of LELC of the bladder with a specific management consisting of a conservative approach even if the tumor was invading the bladder muscle to consolidate literature data.

This represents a particularity of lymphoepithelioma-like carcinoma of the bladder (LELCB) with literature data exposing a favorable outcome in patients with LECLB managed conservatively by means of transurethral resection of bladder tumor (TURBT), chemotherapy, or radiotherapy.

## 2. Case Presentation

A 63-year-old patient presents to our department complaining of macroscopic hematuria occurring for 2 months. She had no prior personal or familial medical history of this complaint.

**Figure 1:**
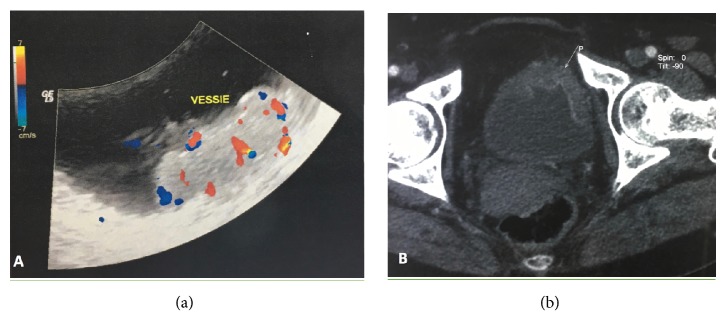
Preoperative imaging of the bladder lesion. (a) Ultrasonography showing vascularized parietal lesion. (b) Scanographic aspect of the bladder tumor.

An abdominopelvic Doppler ultrasound showed a vascularized parietal lesion of the left lateral bladder wall measuring 2 centimeters with a normal upper urinary tract (Figure 1(a)).

A chest and abdominopelvic computed tomography did not find neither hydronephrosis and lymph node involvement, nor a distant metastasis, but a suspected lesion in the vertebral body of D10.

The patient underwent a cystoscopy, which revealed a unique non-papillary large tumor of solid morphology, located on the left lateral wall of the bladder. An extensive transurethral resection of the bladder tumor (TURBT) in a single session was performed.

Postoperative recovery was uneventful.

**Figure 2:**
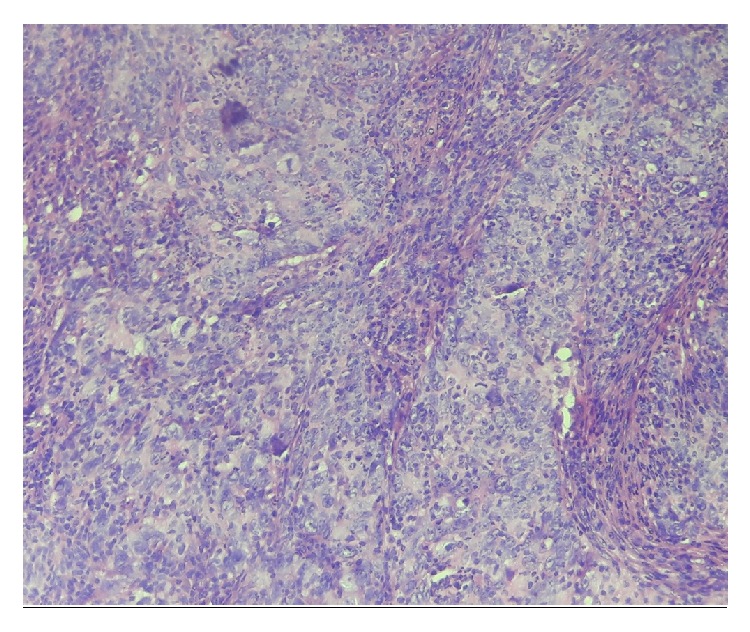
Microscopic aspect of lymphoepithelial-like carcinoma of the bladder.

Histological study revealed a urothelial mucosa infiltrated by tumor proliferation made of nests and ranges of large-sized cells with eosinophilic cytoplasm and frankly atypical and nucleolated nuclei. Muscle layers were also invaded by this proliferation and the tumor stroma was loaded by lymphoid elements (Figure 2).

**Figure 3:**
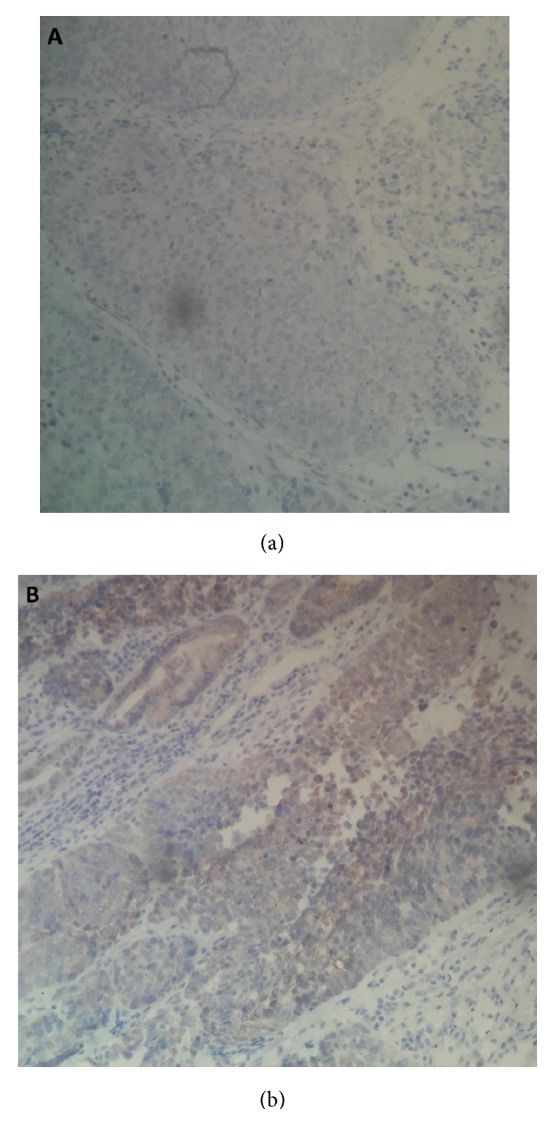
Immunohistochemical staining. (a) negative staining for CD7 and CD20. (b) positive staining for CKAE1/AE3.

On immunohistochemical study, this proliferation was positively stained by epithelial markers(AE1/AE3) but negatively stained for (CK7 and CK20). The final histopathological diagnosis was pure type of LELCB (Figure 3).

For the suspected lesion of the vertebral body of D10 the bone scintigraphy did not find any abnormal results.

**Figure 4:**
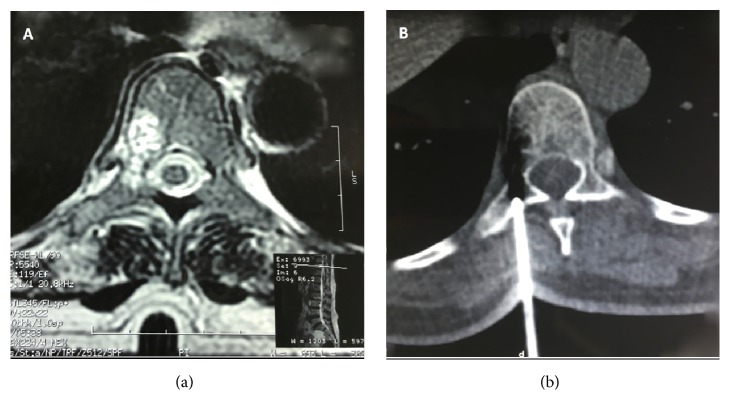
(a) MRI showing an abnormal signal blowing the cortical of the D10 vertebral body. (b) scan guided biopsy of the suspected lesion.

Magnetic resonance imaging showed high intense signal in T1 and T2 of the D10 vertebral body lesion with important enhancement after gadolinium injection which was in favor of vertebral angioma. That was confirmed by histological findings of a scan guided biopsy of the lesion (Figure 4).

A second TURB was performed one month later and did not show any malignancy. A deep resection of the former tumor site was done. Histological examination of the transurethral resection specimen showed a nonspecific inflammatory rearrangement.

Since the histological finding shows the existence of pure LELC, the extension study found no other evident locations of malignancy and given that the patient sought organ-conservation approaches and refused radiotherapy fear of its side effects and that her general condition was very good that can withstand systemic chemotherapy, a decision with the oncological department was reached to begin chemotherapy with gemcitabine combined with cisplatin even if the tumor was invading the muscle.

Chemotherapy sessions were uneventful. Recent imaging and cystoscopic investigations have confirmed disease remission with no findings suggestive of tumor recurrence.

The patient has been under close clinical and radiological follow-up of 17 months without recurrence.

## 3. Discussion

The term lymphoepithelioma refers to an undifferentiated nasopharyngeal carcinoma, characterized by epithelial neoplastic cells growing in solid or discohesive sheets intermingled with a prominent lymphoid infiltrate [5].

Carcinomas with similar histological features arising outside the nasopharynx have been called lymphoepithelioma-like carcinomas and have been reported in other organs including the prostate, breast, uterine cervix, skin, lung, trachea, thymus, stomach and salivary glands [6–8].

The reported incidence of LELCB is 0.3%-1.3% of all bladder cancers, with 144 cases reported in the English literature till March 2018.

According to a recent exhaustive review of LELCB in English literature published in 2017 by Yang et al. reporting 140 cases of LELCB published between 1991 and 2016 the tumor usually occurs in adulthood with a male predominance. In this review the chief complaint was gross hematuria constantly present. The stage at diagnosis of those tumors was in 87% of cases T2–T3 stage [8].

Till now the exact pathogenesis of this tumor is not well established. Although the association between Epstein-Barr virus and lymphoepithelioma of the nasopharynx and other tissues (lung, stomach, thymus, and salivary gland) is already proved, in the bladder, the hybridization with Epstein-Barr virus encoded RNA has been reported to be consistently negative in different series [6, 9, 10].

Abnormalities of p53 regulation were also suggested for having a role in the pathogenesis of LELCB [11].

Some authors reported the recurrence of urothelial carcinoma treated with BCG therapy in a lymphoepithelial mode without mentioning a link between BCG therapy and LELCB [11–13].

Only Gastaud et al. suggested the role of BCG therapy in the activation of the lymphoid system based on publications of the mechanisms of action of BCG therapy [13].

Differentiation of LELCB from urothelial carcinoma is important, since it has implications for prognosis and treatment.

According to the WHO classification criteria, LELCB is defined as a subtype of undifferentiated carcinomas. Histologically, the tumor is composed of nests, sheets, and cords of undifferentiated cells with large pleomorphic nuclei, prominent nucleoli, indistinct cytoplasmic borders, and a syncytial appearance. The background consists of a prominent lymphoid stroma that includes T and B lymphocytes, plasma cells, histiocytes, and occasional neutrophils or eosinophils [14].

Based on the proportion of lymphoepithelioma component Amin et al. described a classification system; tumors are categorized as pure (all component is lymphoepithelial elements), predominant (more than a half) or focal (less than a half), with the three types determining a patient's prognosis [10].

Immunohistochemical stains are helpful in distinguishing between LELCB and its differential diagnosis especially primary lymphoma of the bladder.

The particularity of this variant differentiation of urothelial carcinoma is its relatively favorable prognosis compared to pure urothelial carcinoma especially the pure and predominant forms.

This has been explicated by several authors who analyzed the lymphoid inflammatory component in LELCB by the importance of the inflammatory infiltrate and cytotoxic T-lymphocytes reflecting the strong immune response against the tumor cells and potentiating the effect of chemotherapy and radiotherapy [10, 11, 15].

Izquierdo-García et al. suggested that the remarkable response of cytotoxic T-lymphocytes in LELCB may be related to a better survival in some tumors and may represent an independent prognostic factor [11].

The sensitivity of nasopharynx lymphoepithelioma to chemotherapy and radiotherapy inspired their use in LELCB.

Till now the appropriate management of LELC of the bladder is not unified and as for the other variant of urothelial carcinoma there are no clear guidelines about LELCB.

The existing therapeutic procedures are TURBT, partial cystectomy, and radical cystectomy for primary treatments while adjuvant treatments include systemic chemotherapy, radiotherapy, combined chemotherapy and radiotherapy, and intravesical chemotherapy and as reported in the review of Rodríguez-Cabello et al., with a favorable outcome, disease-free survival rate was higher than 80% in the pure and predominant forms confirming their good prognosis [16].

This good result of chemotherapy and radiotherapy in LELCB allowed bladder preservation therapy even in case of locally advanced tumors instead of radical cystectomy that usually involves the uterus in women (anterior pelvectomy) damaging life quality especially in young women.

Yoshino et al. reported 13 cases treated with transurethral resection of bladder tumor (TURBT) and chemotherapy: only 9 pure and 2 predominant and 2 mixed (predominant or focal) with a mean follow-up of 37 mounts (72-2 mounts) with no evidence of disease when published (Table 1). This literature data shows that a combination of TURB and adjuvant chemotherapy is probably effective against pure or predominant LELCB [17].

A variety of chemotherapy regimens have been used MVAC (methotrexate, vinblastine, doxorubicin, and cisplatin) or GC (gemcitabine and cisplatin), with a promising outcome for platinum-based agents.

In the light of the existing data illustrating the good outcome of pure and predominant LELCB even if invasive, salvage bladder therapy including TURBT resecting deep muscle layers and adjuvant systemic chemotherapy can be adopted as the first therapeutic option permitting, especially in women, preserving an intact body pattern and to provide a significant quality of life gain compared to replacement or urinary diversion techniques.

Pure and predominant LELCB seems to be a gentle variant of urothelial carcinoma

Correct histological diagnosis specifying the percentage of each component is very important since it has therapeutic and prognostic implications.

Chemotherapy is an important therapeutic arsenal in LELCB and should be undertaken, since patients with LELCB present an early onset of symptoms with a preserved state of health enabling them to support systemic chemotherapy.

More studies and experiences in the management of this rare urothelial variant are needed to establish clear guidelines of this entity.

## Figures and Tables

**Table 1:** Characteristics and outcome of patients treated with TURBT and systemic chemotherapy [4]. TURBT: transurethral resection of bladder tumor. NED: no evidence of disease.   CH: systemic chemotherapy.

N	First author	Sex	Age	Classification	Stage	Treatment	Follow up	Outcome
1	Dinney	M	52	Pure	T2	TURBT ,CH	72	NED
	1993							

2	Dinney	M	68	Pure	T2	TURBT ,CH	60	NED
	1993							

3	Dinney	M	63	Pure	T2	TURBT ,CH	11	NED
	1993							

4	Amine	M	52	Pure	T2	TURBT ,CH	72	NED
	1991							

5	Amine	M	68	Pure	T2	TURBT ,CH	60	NED
	1991							

6	Amine	M	63	Pure	T2	TURBT ,CH	11	NED
	1991							

7	Amine	F	71	Predominent	T2	TURBT ,CH	9	NED
	1991							

8	Tamas	-	-	Mixed	T1	TURBT ,CH	6	NED
	2007							

9	Tamas	-	-	Mixed	T2	TURBT ,CH	2	NED
	2007							

10	Constantinides	M	_	Pure	T2	TURBT ,CH	34	NED
	2001							

11	Lopez-B	F	69	Pure	T2	TURBT ,CH	21	NED
	2001							

12	Lopez-B	M	81	Pure	T2	TURBT,CH	47	NED
	2001							

13	Pantelides	M	64	Predominant	T2	TURBT ,CH	6	NED
	2012							

14	**Our case**	**F**	**63**	**Pure**	**T2**	**TURBT ,CH**	**17**	**NED**
	**2018**							

## Abbreviations

LELC: Lymphoepithelioma-like carcinoma
LELBC: Lymphoepithelioma-like carcinoma of the bladder
TURBT: Transurethral resection of bladder tumor.

## References

[1] Ferlay J., Soerjomataram I., Dikshit R. (2015). Cancer incidence and mortality worldwide: sources, methods and major patterns in GLOBOCAN 2012. *International Journal of Cancer*.

[2] Xylinas E., Rink M., Robinson B. D. (2013). Impact of histological variants on oncological outcomes of patients with urothelial carcinoma of the bladder treated with radical cystectomy. *European Journal of Cancer*.

[3] Humphrey P. A., Moch H., Cubilla A. L., Ulbright T. M., Reuter V. E. (2016). The 2016 WHO Classification of Tumours of the Urinary System and Male Genital Organs—Part B: Prostate and Bladder Tumours. *European Urology*.

[4] Chaker K., Sellami A., Ouanes Y. (2018). About a case of lymphoepithelioma-like carcinoma of the bladder. *Urology Case Reports*.

[5] Carbone A., Micheau C. (1982). Pitfalls in microscopic diagnosis of undifferentiate carcinoma of nasopharyngeal type (lymphoepithelioma). *Cancer*.

[6] Tamas E. F., Nielsen M. E., Schoenberg M. P., Epstein J. I. (2007). Lymphoepithelioma-like carcinoma of the urinary tract: A clinicopathological study of 30 pure and mixed cases. *Modern Pathology*.

[7] Chou C., Yang S., Tzen C. (2012). Primary lymphoepithelioma-like carcinoma of the urinary bladder: Case report and literature review. *Urological Science*.

[8] Yang A. W., Pooli A., Lele S. M., Kim I. W., Davies J. D., LaGrange C. A. (2017). Lymphoepithelioma-like, a variant of urothelial carcinoma of the urinary bladder: a case report and systematic review for optimal treatment modality for disease-free survival. *BMC Urology*.

[9] Gulley M. L., Amin M. B., Nicholls J. M. (1995). Epstein-Barr virus is detected in undifferentiated nasopharyngeal carcinoma but not in lymphoepithelioma-like carcinoma of the urinary bladder. *Human Pathology*.

[10] Amin M. B., Ro J. Y., Lee K. M. (1994). Lymphoepithelioma-like carcinoma of the urinary bladder. *The American Journal of Surgical Pathology*.

[11] Izquierdo-Garcia F. M., Garcia-Diez F., Fernandez I. (2004). Lymphoepithelioma—like carcinoma of the bladder: three cases with clinicopathologic and p53 protein expression study. *Virchow Arch*.

[12] Mayer E. K., Beckley I., Winkler M. H. (2007). Lymphoepithelioma-like carcinoma of the urinary bladder - Diagnostic and clinical implications. *Nature Clinical Practice Urology*.

[13] Gastaud O., Demailly M., Guilbert E., Colombat M., Petit J. (2002). Lympho-epithelioma of the bladder. *Progres En Urologie*.

[14] Moch H., Humphrey P. A., Ulbright T. M., Reuter V. E. (2016). World health organization classification of tumours. *Pathology and Genetics of Tumours of the Urinary System and Male Genital Organs*.

[15] Bueno Serrano G., Arias Fúnez F., González López R. (2008). Carcinoma vesical linfoepitelioma-like: revisión de la literatura y aportación de un nuevo caso. *Archivos Españoles de Urología*.

[16] Rodríguez-Cabello M. A., Méndez-Rubio S., Sanz-Miguelañez J. L. (2017). Lymphoepithelioma-Like Bladder Carcinoma: A Diagnostic and Therapeutic Challenge. Contribution Using a New Case and Review of the Literature. *Clinical Genitourinary Cancer*.

[17] Yoshino T., Ohara S., Moriyama H. (2014). Lymphoepithelioma-like carcinoma of the urinary bladder: A case report and review of the literature. *BMC Research Notes*.

